# Supplementary material for: The Cac1 subunit of histone chaperone CAF-1 organizes CAF-1-H3/H4 architecture and tetramerizes histones
Source: eLife. 2016 Sep 30;5:e18023. doi: 10.7554/eLife.18023 (PMC5045291; doi:10.7554/eLife.18023)
Supplement: Supplementary file 1. — (A) Peptides identified in HX studies. (B) Primers used in the studies in yeast. (C) Chemically cross-linked peptides identified by XL-MS. (D) Cac1C putative dimer contacts. DOI: http://dx.doi.org/10.7554/eLife.18023.020 [file elife-18023-supp1.docx]

**Table 1A.** Peptides identified in HX studies

|  |  |  |  |  | **D_2_O Incorporated**  **(CAF-1 or H3/H4 alone)** | | **D_2_O Incorporated**  **(CAF-1-H3/H4)** | | |
| --- | --- | --- | --- | --- | --- | --- | --- | --- | --- |
| **Sub-unit** | **Amino  Acids** | **Peptide Sequence** | **#  Exchan.  Amides** | **Peptide  Mass** | **30'** | **60'** | **30'** | **60'** | |
| Cac1 | 4-22 | HLKSIPLQDDTKKKGILSF | 18 | 2168.24 | 6.18 | 6.24 | 6.18 | 6.39 | |
| Cac1 | 6-22 | KSIPLQDDTKKKGILSF | 16 | 1918.10 | 5.91 | 5.78 | 6.07 | 6.38 | |
| Cac1 | 23-34 | FQNTTTVKSNKF | 12 | 1414.73 | 4.95 | 4.91 | 4.84 | 5.18 | |
| Cac1 | 41-57 | VITLDDPKEDVSGPMIE | 15 | 1857.92 | 5.22 | 5.25 | 5.15 | 5.46 | |
| Cac1 | 44-57 | LDDPKEDVSGPMIE | 12 | 1544.71 | 1.25 | 1.39 | 4.24 | 4.48 | |
| Cac1 | 50-57 | DVSGPMIE | 7 | 847.39 | 2.79 | 2.83 | 2.64 | 2.93 | |
| Cac1 | 216-233 | IRLKEEAKERAQSRIGNF | 18 | 2145.19 | 8.63 | 8.47 | 8.30 | 8.68 | |
| Cac1 | 234-246 | FKKLSDSNTPVVE | 12 | 1463.77 | 3.96 | 3.91 | 3.92 | 4.14 | |
| Cac1 | 256-276 | PFYAKDGVRVSNKWKLTKVEL | 20 | 2478.39 | 8.50 | 8.62 | 8.04 | 8.63 | |
| Cac1 | 277-287 | EGSKRKIDDEL | 11 | 1289.67 | 0.92 | 1.24 | 0.86 | 1.05 | |
| Cac1 | 288-300 | LNSKDKTSSDDLL | 13 | 1435.73 | 2.36 | 2.50 | 2.35 | 2.59 | |
| Cac1 | 304-322 | QSRRLPRGHKIKRKAVDVL | 18 | 2257.38 | 5.49 | 5.80 | 3.61 | 4.13 | |
| Cac1 | 321-335 | VLQQMPLKEKTDDEL | 14 | 1786.93 | 4.10 | 4.03 | 4.54 | 4.64 | |
| Cac1 | 323-335 | QQMPLKEKTDDEL | 12 | 1574.77 | 3.42 | 3.31 | 3.55 | 3.75 | |
| Cac1 | 339-349 | LAQVPHKYIKF | 10 | 1343.78 | 0.75 | 1.06 | 0.54 | 0.70 | |
| Cac1 | 340-349 | AQVPHKYIKF | 9 | 1230.70 | 0.91 | 1.10 | 0.38 | 0.40 | |
| Cac1 | 343-349 | PHKYIKF | 6 | 932.54 | 0.54 | 0.67 | 0.39 | 0.43 | |
| Cac1 | 350-360 | YENVRPPFIGT | 9 | 1292.66 | 1.90 | 2.13 | 1.36 | 1.55 | |
| Cac1 | 352-360 | NVRPPFIGT | 7 | 1000.56 | 1.01 | 1.30 | 0.70 | 0.80 | |
| Cac1 | 365-380 | FTLPPNDPFSTKGTGF | 13 | 1725.85 | 3.30 | 3.55 | 2.97 | 3.39 | |
| Cac1 | 365-381 | FTLPPNDPFSTKGTGFN | 14 | 1839.89 | 3.67 | 3.93 | 3.36 | 3.87 | |
| Cac1 | 456-462 | KSNFENL | 7 | 851.43 | 2.02 | 2.00 | 1.86 | 2.09 | |
| Cac1 | 463-473 | SEENKRYLQQL | 11 | 1407.72 | 1.43 | 1.65 | 0.44 | 0.54 | |
| Cac1 | 476-496 | EVIIETDGPIDPFKEPKTSSL | 18 | 2315.20 | 6.30 | 6.37 | 6.18 | 6.70 | |
| Cac1 | 476-505 | EVIIETDGPIDPFKEPKTSSLPSKRSNSDL | 26 | 3299.70 | 7.56 | 7.38 | 7.54 | 8.02 | |
| Cac1 | 478-496 | IIETDGPIDPFKEPKTSSL | 16 | 2087.09 | 4.93 | 4.99 | 4.90 | 5.19 | |
| Cac1 | 478-505 | IIETDGPIDPFKEPKTSSLPSKRSNSDL | 24 | 3071.59 | 6.01 | 5.90 | 6.17 | 6.51 | |
| Cac1 | 523-530 | MITDPMDL | 7 | 935.42 | 1.41 | 1.52 | 1.43 | 1.60 | |
| Cac1 | 531-538 | LRLFDGVQ | 8 | 947.53 | 1.17 | 1.35 | 1.09 | 1.26 | |
| Cac1 | 531-542 | LRLFDGVQDSTF | 12 | 1397.71 | 2.20 | 2.38 | 2.00 | 2.28 | |
| Cac1 | 534-542 | FDGVQDSTF | 9 | 1015.44 | 1.91 | 2.04 | 1.88 | 2.10 | |
| Cac1 | 543-549 | SLGTVTE | 7 | 706.36 | 1.93 | 2.17 | 1.69 | 2.02 | |
| Cac1 | 550-569 | IAQKNLPQYNKQTIKNTIKE | 19 | 2372.33 | 4.02 | 4.48 | 5.01 | 6.18 | |
| Cac1 | 551-569 | AQKNLPQYNKQTIKNTIKE | 18 | 2259.25 | 3.77 | 4.16 | 4.78 | 6.01 | |
| Cac1 | 556-569 | PQYNKQTIKNTIKE | 13 | 1704.93 | 2.86 | 3.10 | 3.50 | 4.57 | |
| Cac1 | 570-591 | YAIRSSGKGDLPRKWVIKDAQN | 21 | 2502.36 | 5.42 | 5.63 | 5.94 | 6.68 | |
| Cac1 | 596-608 | RANANMPTPSLSG | 11 | 1315.64 | 4.33 | 4.23 | 4.61 | 4.71 | |
| Cac2 | 7-17 | QIYWHDSQPVY | 11 | 1435.66 | 1.56 | 1.57 | 0.77 | 0.98 | |
| Cac2 | 7-20 | QIYWHDSQPVYSLT | 14 | 1736.83 | 1.40 | 1.49 | 0.72 | 0.80 | |
| Cac2 | 31-44 | FTAGGDNKVRIWKL | 14 | 1604.89 | 0.95 | 1.34 | 0.69 | 0.85 | |
| Cac2 | 34-44 | GGDNKVRIWKL | 11 | 1285.74 | 0.63 | 0.96 | 0.63 | 0.84 | |
| Cac2 | 62-75 | DFLGSLTHHEQAIN | 14 | 1581.77 | 2.27 | 2.32 | 1.63 | 1.89 | |
| Cac2 | 62-76 | DFLGSLTHHEQAINV | 15 | 1680.83 | 1.99 | 2.16 | 1.62 | 1.84 | |
| Cac2 | 63-76 | FLGSLTHHEQAINV | 14 | 1565.81 | 1.23 | 1.31 | 1.18 | 1.30 | |
| Cac2 | 76-86 | VIRFNSKGDVL | 11 | 1247.71 | 1.64 | 1.75 | 1.59 | 1.99 | |
| Cac2 | 77-86 | IRFNSKGDVL | 10 | 1148.64 | 1.55 | 1.50 | 1.53 | 1.71 | |
| Cac2 | 87-97 | ASAGDDGQVLL | 11 | 1045.52 | 2.01 | 2.29 | 1.66 | 2.09 | |
| Cac2 | 109-117 | SVVRPFGMD | 9 | 1007.50 | 3.31 | 3.24 | 3.17 | 3.30 | |
| Cac2 | 110-117 | VVRPFGMD | 8 | 920.47 | 2.49 | 2.45 | 2.48 | 2.57 | |
| Cac2 | 118-130 | AETSEADENKEKW | 13 | 1536.68 | 3.90 | 3.82 | 4.08 | 4.30 | |
| Cac2 | 118-147 | AETSEADENKEKWVVWKRLRGGSGATAAAE | 30 | 3246.61 | 9.04 | 9.02 | 9.04 | 9.65 | |
| Cac2 | 120-147 | TSEADENKEKWVVWKRLRGGSGATAAAE | 28 | 3046.53 | 8.21 | 8.19 | 7.86 | 8.28 | |
| Cac2 | 148-159 | IYDLAWSPDNRN | 12 | 1463.69 | 0.83 | 1.08 | 0.65 | 0.83 | |
| Cac2 | 149-159 | YDLAWSPDNRN | 11 | 1350.61 | 0.69 | 0.92 | 0.70 | 0.99 | |
| Cac2 | 151-159 | LAWSPDNRN | 9 | 1072.52 | 1.07 | 1.33 | 0.78 | 1.05 | |
| Cac2 | 152-159 | AWSPDNRN | 8 | 959.43 | 1.40 | 1.63 | 1.18 | 1.58 | |
| Cac2 | 172-178 | FDVGAGM | 7 | 696.30 | 2.24 | 2.29 | 1.92 | 2.23 | |
| Cac2 | 172-179 | FDVGAGML | 8 | 809.39 | 2.73 | 2.76 | 2.50 | 2.81 | |
| Cac2 | 179-200 | LVCGQSDHGHYVQGVAWDPLNQ | 22 | 2423.12 | 0.91 | 1.19 | 1.00 | 1.27 | |
| Cac2 | 180-200 | VCGQSDHGHYVQGVAWDPLNQ | 21 | 2310.04 | 0.81 | 1.09 | 0.83 | 1.07 | |
| Cac2 | 180-201 | VCGQSDHGHYVQGVAWDPLNQF | 22 | 2457.10 | 0.58 | 0.87 | 0.60 | 0.81 | |
| Cac2 | 193-200 | VAWDPLNQ | 8 | 942.47 | 0.53 | 0.66 | 0.39 | 0.54 | |
| Cac2 | 193-201 | VAWDPLNQF | 9 | 1089.54 | 0.56 | 0.75 | 0.34 | 0.50 | |
| Cac2 | 201-213 | FILSQSADRSLHV | 13 | 1472.79 | 1.13 | 1.35 | 0.33 | 0.39 | |
| Cac2 | 201-215 | FILSQSADRSLHVYG | 15 | 1692.87 | 1.32 | 1.47 | 0.49 | 0.53 | |
| Cac2 | 201-218 | FILSQSADRSLHVYGVIL | 18 | 2018.11 | 1.76 | 2.10 | 0.82 | 0.97 | |
| Cac2 | 202-218 | ILSQSADRSLHVYGVIL | 17 | 1871.04 | 2.06 | 2.29 | 1.00 | 1.20 | |
| Cac2 | 246-256 | RTNYLFHNETL | 11 | 1407.70 | 2.42 | 2.60 | 1.52 | 1.69 | |
| Cac2 | 251-259 | FHNETLPSF | 9 | 1091.52 | 2.46 | 2.39 | 0.83 | 1.01 | |
| Cac2 | 260-271 | FRRCSISPCGGL | 12 | 1295.63 | 1.51 | 1.58 | 1.49 | 1.66 | |
| Cac2 | 272-278 | VVIPSGV | 7 | 670.41 | 1.20 | 1.28 | 0.13 | 0.16 | |
| Cac2 | 279-289 | YKVAGDEVANC | 11 | 1168.53 | 4.58 | 4.59 | 4.61 | 4.86 | |
| Cac2 | 290-299 | VYVYTRSGIL | 10 | 1170.65 | 0.70 | 0.89 | 0.84 | 1.02 | |
| Cac2 | 290-322 | VYVYTRSGILNSAGGVKNRPAIRIPSLKKPALM | 33 | 3570.05 | 5.23 | 5.44 | 4.80 | 5.38 | |
| Cac2 | 300-321 | NSAGGVKNRPAIRIPSLKKPAL | 22 | 2287.37 | 4.50 | 4.68 | 3.04 | 3.36 | |
| Cac2 | 300-322 | NSAGGVKNRPAIRIPSLKKPALM | 23 | 2418.41 | 4.28 | 4.42 | 3.00 | 3.33 | |
| Cac2 | 302-322 | AGGVKNRPAIRIPSLKKPALM | 21 | 2217.34 | 4.01 | 4.08 | 2.47 | 2.80 | |
| Cac2 | 322-329 | MAAFSPVF | 8 | 869.42 | 0.42 | 0.47 | 0.36 | 0.42 | |
| Cac2 | 323-329 | AAFSPVF | 7 | 738.38 | 0.47 | 0.50 | 0.45 | 0.49 | |
| Cac2 | 330-346 | YETCQKSVLKLPYKLVF | 17 | 2059.13 | 2.97 | 3.06 | 2.88 | 3.30 | |
| Cac2 | 332-346 | TCQKSVLKLPYKLVF | 15 | 1767.02 | 2.82 | 2.73 | 2.58 | 3.00 | |
| Cac2 | 335-346 | KSVLKLPYKLVF | 12 | 1434.91 | 2.26 | 2.38 | 1.83 | 2.13 | |
| Cac2 | 347-355 | AIATTNEVL | 9 | 931.51 | 0.65 | 0.72 | 0.39 | 0.50 | |
| Cac2 | 356-367 | VYDTDVLEPLCV | 12 | 1365.66 | 1.07 | 1.29 | 0.81 | 1.04 | |
| Cac2 | 360-367 | DVLEPLCV | 8 | 887.45 | 0.77 | 0.86 | 0.60 | 0.66 | |
| Cac2 | 361-366 | VLEPLC | 6 | 673.36 | 0.04 | 0.13 | 0.14 | 0.19 | |
| Cac2 | 361-367 | VLEPLCV | 7 | 772.43 | 0.16 | 0.23 | 0.08 | 0.03 | |
| Cac2 | 367-378 | VVGNIHYSPITD | 12 | 1314.67 | 0.81 | 0.87 | 0.69 | 0.75 | |
| Cac2 | 367-379 | VVGNIHYSPITDL | 13 | 1427.75 | 0.59 | 0.71 | 0.55 | 0.59 | |
| Cac2 | 368-378 | VGNIHYSPITD | 11 | 1215.60 | 0.46 | 0.55 | 0.34 | 0.40 | |
| Cac2 | 368-379 | VGNIHYSPITDL | 12 | 1328.68 | 0.29 | 0.42 | 0.26 | 0.28 | |
| Cac2 | 380-389 | AWSEDGSTLL | 10 | 1078.51 | 0.65 | 0.60 | 0.63 | 0.69 | |
| Cac2 | 389-396 | LISSTDGF | 8 | 839.41 | 0.50 | 0.53 | 0.24 | 0.23 | |
| Cac2 | 390-396 | ISSTDGF | 7 | 726.33 | 0.51 | 0.63 | 0.22 | 0.24 | |
| Cac2 | 400-417 | VSIDTETQFGSRIEPPAM | 18 | 1977.96 | 5.33 | 5.35 | 4.90 | 5.26 | |
| Cac2 | 409-417 | GSRIEPPAM | 9 | 957.48 | 1.98 | 1.97 | 1.94 | 1.98 | |
| Cac2 | 418-426 | HAEPLDTDE | 8 | 1026.44 | 1.67 | 1.63 | 1.73 | 1.83 | |
| Cac2 | 427-443 | SAVAAKNQREAGGIVNM | 17 | 1715.89 | 7.64 | 7.65 | 7.85 | 8.28 | |
| Cac3 | 12-19 | ASSIPIDL | 7 | 815.45 | 1.95 | 1.91 | 1.80 | 1.94 | |
| Cac3 | 33-37 | LYDYL | 5 | 686.34 | 0.10 | 0.16 | 0.09 | 0.08 | |
| Cac3 | 36-47 | YLNTNSTKWPSL | 11 | 1423.72 | 1.39 | 1.55 | 1.03 | 1.18 | |
| Cac3 | 38-47 | NTNSTKWPSL | 9 | 1147.57 | 1.35 | 1.48 | 1.03 | 1.21 | |
| Cac3 | 40-47 | NSTKWPSL | 7 | 932.48 | 1.29 | 1.40 | 0.72 | 0.87 | |
| Cac3 | 52-57 | FPDLDT | 5 | 707.32 | 0.99 | 1.08 | 0.99 | 1.01 | |
| Cac3 | 62-66 | HRILL | 5 | 651.43 | 0.01 | 0.08 | 0.13 | 0.12 | |
| Cac3 | 62-68 | HRILLSS | 7 | 825.49 | 0.18 | 0.25 | 0.28 | 0.20 | |
| Cac3 | 67-81 | SSFTSSQKPEDETIY | 14 | 1718.78 | 1.61 | 1.73 | 1.27 | 1.54 | |
| Cac3 | 70-81 | TSSQKPEDETIY | 11 | 1397.64 | 1.40 | 1.49 | 1.24 | 1.49 | |
| Cac3 | 80-96 | IYISKISTLGHIKWSSL | 17 | 1946.11 | 2.62 | 2.72 | 2.38 | 2.79 | |
| Cac3 | 82-96 | ISKISTLGHIKWSSL | 15 | 1669.96 | 2.59 | 2.72 | 2.50 | 2.83 | |
| Cac3 | 82-99 | ISKISTLGHIKWSSLNNF | 18 | 2045.12 | 3.54 | 3.34 | 2.90 | 3.54 | |
| Cac3 | 85-96 | ISTLGHIKWSSL | 12 | 1341.75 | 1.88 | 1.91 | 1.98 | 2.18 | |
| Cac3 | 104-122 | MEFKPENSTRFPSKHLVND | 17 | 2276.11 | 3.02 | 3.09 | 3.11 | 3.33 | |
| Cac3 | 123-146 | ISIFFPNGECNRARYLPQNPDIIA | 21 | 2748.39 | 2.08 | 2.33 | 1.92 | 2.15 | |
| Cac3 | 125-146 | IFFPNGECNRARYLPQNPDIIA | 19 | 2548.28 | 1.25 | 1.48 | 1.24 | 1.52 | |
| Cac3 | 127-146 | FPNGECNRARYLPQNPDIIA | 17 | 2288.12 | 1.48 | 1.68 | 1.18 | 1.47 | |
| Cac3 | 133-153 | NRARYLPQNPDIIAGASSDGA | 19 | 2186.09 | 1.44 | 1.69 | 1.62 | 1.83 | |
| Cac3 | 147-155 | GASSDGAIY | 9 | 840.37 | 0.51 | 0.55 | 0.59 | 0.65 | |
| Cac3 | 181-189 | FGSHGVIQD | 9 | 959.46 | 3.05 | 3.03 | 2.68 | 2.77 | |
| Cac3 | 181-192 | FGSHGVIQDVEA | 12 | 1258.61 | 4.50 | 4.27 | 4.35 | 4.59 | |
| Cac3 | 193-202 | MDTSSADINE | 10 | 1082.43 | 3.63 | 3.49 | 3.58 | 3.79 | |
| Cac3 | 207-212 | AWNLQQ | 6 | 759.38 | 0.81 | 0.83 | 0.75 | 0.82 | |
| Cac3 | 211-216 | QQEALL | 6 | 701.38 | 0.84 | 0.90 | 0.60 | 0.68 | |
| Cac3 | 217-232 | LSSHSNGQVQVWDIKQ | 16 | 1825.92 | 1.06 | 1.23 | 0.98 | 1.19 | |
| Cac3 | 229-242 | DIKQYSHENPIIDL | 13 | 1684.85 | 2.82 | 3.22 | 2.42 | 3.01 | |
| Cac3 | 229-244 | DIKQYSHENPIIDLPL | 14 | 1894.99 | 2.45 | 2.81 | 2.50 | 3.00 | |
| Cac3 | 233-242 | YSHENPIIDL | 9 | 1200.59 | 1.05 | 1.46 | 0.97 | 1.27 | |
| Cac3 | 233-244 | YSHENPIIDLPL | 10 | 1410.73 | 1.07 | 1.36 | 0.93 | 1.23 | |
| Cac3 | 245-256 | VSINSDGTAVND | 12 | 1191.55 | 3.43 | 3.54 | 3.39 | 3.72 | |
| Cac3 | 257-266 | VTWMPTHDSL | 9 | 1186.56 | 1.12 | 1.08 | 1.15 | 1.21 | |
| Cac3 | 279-304 | LDLRTKKEKLQSNREKHDGGVNSCRF | 26 | 3058.60 | 4.24 | 4.06 | 4.58 | 5.04 | |
| Cac3 | 305-312 | NYKNSLIL | 8 | 964.55 | 0.54 | 0.62 | 0.57 | 0.64 | |
| Cac3 | 313-323 | ASADSNGRLNL | 11 | 1117.56 | 0.64 | 0.73 | 0.53 | 0.63 | |
| Cac3 | 324-335 | WDIRNMNKSPIA | 11 | 1444.74 | 2.20 | 2.21 | 2.15 | 2.21 | |
| Cac3 | 324-336 | WDIRNMNKSPIAT | 12 | 1545.78 | 2.84 | 2.85 | 2.79 | 2.96 | |
| Cac3 | 346-352 | LEWSPNF | 6 | 892.42 | 1.53 | 1.51 | 1.49 | 1.74 | |
| Cac3 | 347-352 | EWSPNF | 5 | 779.34 | 1.06 | 1.02 | 0.99 | 1.04 | |
| Cac3 | 357-365 | ATAGQEDGL | 9 | 861.39 | 1.31 | 1.44 | 1.20 | 1.33 | |
| Cac3 | 374-389 | EETIFTHGGHMLGVND | 16 | 1756.80 | 1.08 | 1.26 | 1.18 | 1.29 | |
| Cac3 | 375-389 | ETIFTHGGHMLGVND | 15 | 1627.75 | 0.98 | 1.06 | 1.02 | 1.16 | |
| Cac3 | 376-389 | TIFTHGGHMLGVND | 14 | 1498.71 | 1.01 | 1.09 | 1.05 | 1.19 | |
| Cac3 | 389-399 | DISWDAHDPWL | 10 | 1354.61 | 1.13 | 1.29 | 1.18 | 1.47 | |
| Cac3 | 390-399 | ISWDAHDPWL | 9 | 1239.58 | 0.93 | 1.10 | 0.99 | 1.25 | |
| Cac3 | 390-400 | ISWDAHDPWLM | 10 | 1370.62 | 0.99 | 1.14 | 0.92 | 1.15 | |
| Cac3 | 390-401 | ISWDAHDPWLMC | 11 | 1473.63 | 0.87 | 1.05 | 0.88 | 1.09 | |
| Cac3 | 402-418 | SVANDNSVHIWKPAGNL | 16 | 1821.92 | 1.52 | 1.66 | 1.52 | 1.79 | |
| Cac3 | 404-418 | ANDNSVHIWKPAGNL | 14 | 1635.82 | 1.46 | 1.57 | 1.52 | 1.71 | |
|  | |  |  |  |  | **D_2_O Incorporated**  **(CAF-1 or H3/H4 alone)** | | **D_2_O Incorporated**  **(CAF-1-H3/H4)** | |
| **Sub-unit** | | **Amino  Acids** | **Peptide Sequence** | **#  Exchan.  Amides** | **Peptide  Mass** | **30'** | **60'** | **30'** | **60'** |
| Cac1 | | 4-22 | HLKSIPLQDDTKKKGILSF | 18 | 2168.24 | 6.18 | 6.24 | 6.18 | 6.39 |
| Cac1 | | 6-22 | KSIPLQDDTKKKGILSF | 16 | 1918.10 | 5.91 | 5.78 | 6.07 | 6.38 |
| Cac1 | | 23-34 | FQNTTTVKSNKF | 12 | 1414.73 | 4.95 | 4.91 | 4.84 | 5.18 |
| Cac1 | | 41-57 | VITLDDPKEDVSGPMIE | 15 | 1857.92 | 5.22 | 5.25 | 5.15 | 5.46 |
| Cac1 | | 44-57 | LDDPKEDVSGPMIE | 12 | 1544.71 | 1.25 | 1.39 | 4.24 | 4.48 |
| Cac1 | | 50-57 | DVSGPMIE | 7 | 847.39 | 2.79 | 2.83 | 2.64 | 2.93 |
| Cac1 | | 216-233 | IRLKEEAKERAQSRIGNF | 18 | 2145.19 | 8.63 | 8.47 | 8.30 | 8.68 |
| Cac1 | | 234-246 | FKKLSDSNTPVVE | 12 | 1463.77 | 3.96 | 3.91 | 3.92 | 4.14 |
| Cac1 | | 256-276 | PFYAKDGVRVSNKWKLTKVEL | 20 | 2478.39 | 8.50 | 8.62 | 8.04 | 8.63 |
| Cac1 | | 277-287 | EGSKRKIDDEL | 11 | 1289.67 | 0.92 | 1.24 | 0.86 | 1.05 |
| Cac1 | | 288-300 | LNSKDKTSSDDLL | 13 | 1435.73 | 2.36 | 2.50 | 2.35 | 2.59 |
| Cac1 | | 304-322 | QSRRLPRGHKIKRKAVDVL | 18 | 2257.38 | 5.49 | 5.80 | 3.61 | 4.13 |
| Cac1 | | 321-335 | VLQQMPLKEKTDDEL | 14 | 1786.93 | 4.10 | 4.03 | 4.54 | 4.64 |
| Cac1 | | 323-335 | QQMPLKEKTDDEL | 12 | 1574.77 | 3.42 | 3.31 | 3.55 | 3.75 |
| Cac1 | | 339-349 | LAQVPHKYIKF | 10 | 1343.78 | 0.75 | 1.06 | 0.54 | 0.70 |
| Cac1 | | 340-349 | AQVPHKYIKF | 9 | 1230.70 | 0.91 | 1.10 | 0.38 | 0.40 |
| Cac1 | | 343-349 | PHKYIKF | 6 | 932.54 | 0.54 | 0.67 | 0.39 | 0.43 |
| Cac1 | | 350-360 | YENVRPPFIGT | 9 | 1292.66 | 1.90 | 2.13 | 1.36 | 1.55 |
| Cac1 | | 352-360 | NVRPPFIGT | 7 | 1000.56 | 1.01 | 1.30 | 0.70 | 0.80 |
| Cac1 | | 365-380 | FTLPPNDPFSTKGTGF | 13 | 1725.85 | 3.30 | 3.55 | 2.97 | 3.39 |
| Cac1 | | 365-381 | FTLPPNDPFSTKGTGFN | 14 | 1839.89 | 3.67 | 3.93 | 3.36 | 3.87 |
| Cac1 | | 456-462 | KSNFENL | 7 | 851.43 | 2.02 | 2.00 | 1.86 | 2.09 |
| Cac1 | | 463-473 | SEENKRYLQQL | 11 | 1407.72 | 1.43 | 1.65 | 0.44 | 0.54 |
| Cac1 | | 476-496 | EVIIETDGPIDPFKEPKTSSL | 18 | 2315.20 | 6.30 | 6.37 | 6.18 | 6.70 |
| Cac1 | | 476-505 | EVIIETDGPIDPFKEPKTSSLPSKRSNSDL | 26 | 3299.70 | 7.56 | 7.38 | 7.54 | 8.02 |
| Cac1 | | 478-496 | IIETDGPIDPFKEPKTSSL | 16 | 2087.09 | 4.93 | 4.99 | 4.90 | 5.19 |
| Cac1 | | 478-505 | IIETDGPIDPFKEPKTSSLPSKRSNSDL | 24 | 3071.59 | 6.01 | 5.90 | 6.17 | 6.51 |
| Cac1 | | 523-530 | MITDPMDL | 7 | 935.42 | 1.41 | 1.52 | 1.43 | 1.60 |
| Cac1 | | 531-538 | LRLFDGVQ | 8 | 947.53 | 1.17 | 1.35 | 1.09 | 1.26 |
| Cac1 | | 531-542 | LRLFDGVQDSTF | 12 | 1397.71 | 2.20 | 2.38 | 2.00 | 2.28 |
| Cac1 | | 534-542 | FDGVQDSTF | 9 | 1015.44 | 1.91 | 2.04 | 1.88 | 2.10 |
| Cac1 | | 543-549 | SLGTVTE | 7 | 706.36 | 1.93 | 2.17 | 1.69 | 2.02 |
| Cac1 | | 550-569 | IAQKNLPQYNKQTIKNTIKE | 19 | 2372.33 | 4.02 | 4.48 | 5.01 | 6.18 |
| Cac1 | | 551-569 | AQKNLPQYNKQTIKNTIKE | 18 | 2259.25 | 3.77 | 4.16 | 4.78 | 6.01 |
| Cac1 | | 556-569 | PQYNKQTIKNTIKE | 13 | 1704.93 | 2.86 | 3.10 | 3.50 | 4.57 |
| Cac1 | | 570-591 | YAIRSSGKGDLPRKWVIKDAQN | 21 | 2502.36 | 5.42 | 5.63 | 5.94 | 6.68 |
| Cac1 | | 596-608 | RANANMPTPSLSG | 11 | 1315.64 | 4.33 | 4.23 | 4.61 | 4.71 |
| Cac2 | | 7-17 | QIYWHDSQPVY | 11 | 1435.66 | 1.56 | 1.57 | 0.77 | 0.98 |
| Cac2 | | 7-20 | QIYWHDSQPVYSLT | 14 | 1736.83 | 1.40 | 1.49 | 0.72 | 0.80 |
| Cac2 | | 31-44 | FTAGGDNKVRIWKL | 14 | 1604.89 | 0.95 | 1.34 | 0.69 | 0.85 |
| Cac2 | | 34-44 | GGDNKVRIWKL | 11 | 1285.74 | 0.63 | 0.96 | 0.63 | 0.84 |
| Cac2 | | 62-75 | DFLGSLTHHEQAIN | 14 | 1581.77 | 2.27 | 2.32 | 1.63 | 1.89 |
| Cac2 | | 62-76 | DFLGSLTHHEQAINV | 15 | 1680.83 | 1.99 | 2.16 | 1.62 | 1.84 |
| Cac2 | | 63-76 | FLGSLTHHEQAINV | 14 | 1565.81 | 1.23 | 1.31 | 1.18 | 1.30 |
| Cac2 | | 76-86 | VIRFNSKGDVL | 11 | 1247.71 | 1.64 | 1.75 | 1.59 | 1.99 |
| Cac2 | | 77-86 | IRFNSKGDVL | 10 | 1148.64 | 1.55 | 1.50 | 1.53 | 1.71 |
| Cac2 | | 87-97 | ASAGDDGQVLL | 11 | 1045.52 | 2.01 | 2.29 | 1.66 | 2.09 |
| Cac2 | | 109-117 | SVVRPFGMD | 9 | 1007.50 | 3.31 | 3.24 | 3.17 | 3.30 |
| Cac2 | | 110-117 | VVRPFGMD | 8 | 920.47 | 2.49 | 2.45 | 2.48 | 2.57 |
| Cac2 | | 118-130 | AETSEADENKEKW | 13 | 1536.68 | 3.90 | 3.82 | 4.08 | 4.30 |
| Cac2 | | 118-147 | AETSEADENKEKWVVWKRLRGGSGATAAAE | 30 | 3246.61 | 9.04 | 9.02 | 9.04 | 9.65 |
| Cac2 | | 120-147 | TSEADENKEKWVVWKRLRGGSGATAAAE | 28 | 3046.53 | 8.21 | 8.19 | 7.86 | 8.28 |
| Cac2 | | 148-159 | IYDLAWSPDNRN | 12 | 1463.69 | 0.83 | 1.08 | 0.65 | 0.83 |
| Cac2 | | 149-159 | YDLAWSPDNRN | 11 | 1350.61 | 0.69 | 0.92 | 0.70 | 0.99 |
| Cac2 | | 151-159 | LAWSPDNRN | 9 | 1072.52 | 1.07 | 1.33 | 0.78 | 1.05 |
| Cac2 | | 152-159 | AWSPDNRN | 8 | 959.43 | 1.40 | 1.63 | 1.18 | 1.58 |
| Cac2 | | 172-178 | FDVGAGM | 7 | 696.30 | 2.24 | 2.29 | 1.92 | 2.23 |
| Cac2 | | 172-179 | FDVGAGML | 8 | 809.39 | 2.73 | 2.76 | 2.50 | 2.81 |
| Cac2 | | 179-200 | LVCGQSDHGHYVQGVAWDPLNQ | 22 | 2423.12 | 0.91 | 1.19 | 1.00 | 1.27 |
| Cac2 | | 180-200 | VCGQSDHGHYVQGVAWDPLNQ | 21 | 2310.04 | 0.81 | 1.09 | 0.83 | 1.07 |
| Cac2 | | 180-201 | VCGQSDHGHYVQGVAWDPLNQF | 22 | 2457.10 | 0.58 | 0.87 | 0.60 | 0.81 |
| Cac2 | | 193-200 | VAWDPLNQ | 8 | 942.47 | 0.53 | 0.66 | 0.39 | 0.54 |
| Cac2 | | 193-201 | VAWDPLNQF | 9 | 1089.54 | 0.56 | 0.75 | 0.34 | 0.50 |
| Cac2 | | 201-213 | FILSQSADRSLHV | 13 | 1472.79 | 1.13 | 1.35 | 0.33 | 0.39 |
| Cac2 | | 201-215 | FILSQSADRSLHVYG | 15 | 1692.87 | 1.32 | 1.47 | 0.49 | 0.53 |
| Cac2 | | 201-218 | FILSQSADRSLHVYGVIL | 18 | 2018.11 | 1.76 | 2.10 | 0.82 | 0.97 |
| Cac2 | | 202-218 | ILSQSADRSLHVYGVIL | 17 | 1871.04 | 2.06 | 2.29 | 1.00 | 1.20 |
| Cac2 | | 246-256 | RTNYLFHNETL | 11 | 1407.70 | 2.42 | 2.60 | 1.52 | 1.69 |
| Cac2 | | 251-259 | FHNETLPSF | 9 | 1091.52 | 2.46 | 2.39 | 0.83 | 1.01 |
| Cac2 | | 260-271 | FRRCSISPCGGL | 12 | 1295.63 | 1.51 | 1.58 | 1.49 | 1.66 |
| Cac2 | | 272-278 | VVIPSGV | 7 | 670.41 | 1.20 | 1.28 | 0.13 | 0.16 |
| Cac2 | | 279-289 | YKVAGDEVANC | 11 | 1168.53 | 4.58 | 4.59 | 4.61 | 4.86 |
| Cac2 | | 290-299 | VYVYTRSGIL | 10 | 1170.65 | 0.70 | 0.89 | 0.84 | 1.02 |
| Cac2 | | 290-322 | VYVYTRSGILNSAGGVKNRPAIRIPSLKKPALM | 33 | 3570.05 | 5.23 | 5.44 | 4.80 | 5.38 |
| Cac2 | | 300-321 | NSAGGVKNRPAIRIPSLKKPAL | 22 | 2287.37 | 4.50 | 4.68 | 3.04 | 3.36 |
| Cac2 | | 300-322 | NSAGGVKNRPAIRIPSLKKPALM | 23 | 2418.41 | 4.28 | 4.42 | 3.00 | 3.33 |
| Cac2 | | 302-322 | AGGVKNRPAIRIPSLKKPALM | 21 | 2217.34 | 4.01 | 4.08 | 2.47 | 2.80 |
| Cac2 | | 322-329 | MAAFSPVF | 8 | 869.42 | 0.42 | 0.47 | 0.36 | 0.42 |
| Cac2 | | 323-329 | AAFSPVF | 7 | 738.38 | 0.47 | 0.50 | 0.45 | 0.49 |
| Cac2 | | 330-346 | YETCQKSVLKLPYKLVF | 17 | 2059.13 | 2.97 | 3.06 | 2.88 | 3.30 |
| Cac2 | | 332-346 | TCQKSVLKLPYKLVF | 15 | 1767.02 | 2.82 | 2.73 | 2.58 | 3.00 |
| Cac2 | | 335-346 | KSVLKLPYKLVF | 12 | 1434.91 | 2.26 | 2.38 | 1.83 | 2.13 |
| Cac2 | | 347-355 | AIATTNEVL | 9 | 931.51 | 0.65 | 0.72 | 0.39 | 0.50 |
| Cac2 | | 356-367 | VYDTDVLEPLCV | 12 | 1365.66 | 1.07 | 1.29 | 0.81 | 1.04 |
| Cac2 | | 360-367 | DVLEPLCV | 8 | 887.45 | 0.77 | 0.86 | 0.60 | 0.66 |
| Cac2 | | 361-366 | VLEPLC | 6 | 673.36 | 0.04 | 0.13 | 0.14 | 0.19 |
| Cac2 | | 361-367 | VLEPLCV | 7 | 772.43 | 0.16 | 0.23 | 0.08 | 0.03 |
| Cac2 | | 367-378 | VVGNIHYSPITD | 12 | 1314.67 | 0.81 | 0.87 | 0.69 | 0.75 |
| Cac2 | | 367-379 | VVGNIHYSPITDL | 13 | 1427.75 | 0.59 | 0.71 | 0.55 | 0.59 |
| Cac2 | | 368-378 | VGNIHYSPITD | 11 | 1215.60 | 0.46 | 0.55 | 0.34 | 0.40 |
| Cac2 | | 368-379 | VGNIHYSPITDL | 12 | 1328.68 | 0.29 | 0.42 | 0.26 | 0.28 |
| Cac2 | | 380-389 | AWSEDGSTLL | 10 | 1078.51 | 0.65 | 0.60 | 0.63 | 0.69 |
| Cac2 | | 389-396 | LISSTDGF | 8 | 839.41 | 0.50 | 0.53 | 0.24 | 0.23 |
| Cac2 | | 390-396 | ISSTDGF | 7 | 726.33 | 0.51 | 0.63 | 0.22 | 0.24 |
| Cac2 | | 400-417 | VSIDTETQFGSRIEPPAM | 18 | 1977.96 | 5.33 | 5.35 | 4.90 | 5.26 |
| Cac2 | | 409-417 | GSRIEPPAM | 9 | 957.48 | 1.98 | 1.97 | 1.94 | 1.98 |
| Cac2 | | 418-426 | HAEPLDTDE | 8 | 1026.44 | 1.67 | 1.63 | 1.73 | 1.83 |
| Cac2 | | 427-443 | SAVAAKNQREAGGIVNM | 17 | 1715.89 | 7.64 | 7.65 | 7.85 | 8.28 |
| Cac3 | | 12-19 | ASSIPIDL | 7 | 815.45 | 1.95 | 1.91 | 1.80 | 1.94 |
| Cac3 | | 33-37 | LYDYL | 5 | 686.34 | 0.10 | 0.16 | 0.09 | 0.08 |
| Cac3 | | 36-47 | YLNTNSTKWPSL | 11 | 1423.72 | 1.39 | 1.55 | 1.03 | 1.18 |
| Cac3 | | 38-47 | NTNSTKWPSL | 9 | 1147.57 | 1.35 | 1.48 | 1.03 | 1.21 |
| Cac3 | | 40-47 | NSTKWPSL | 7 | 932.48 | 1.29 | 1.40 | 0.72 | 0.87 |
| Cac3 | | 52-57 | FPDLDT | 5 | 707.32 | 0.99 | 1.08 | 0.99 | 1.01 |
| Cac3 | | 62-66 | HRILL | 5 | 651.43 | 0.01 | 0.08 | 0.13 | 0.12 |
| Cac3 | | 62-68 | HRILLSS | 7 | 825.49 | 0.18 | 0.25 | 0.28 | 0.20 |
| Cac3 | | 67-81 | SSFTSSQKPEDETIY | 14 | 1718.78 | 1.61 | 1.73 | 1.27 | 1.54 |
| Cac3 | | 70-81 | TSSQKPEDETIY | 11 | 1397.64 | 1.40 | 1.49 | 1.24 | 1.49 |
| Cac3 | | 80-96 | IYISKISTLGHIKWSSL | 17 | 1946.11 | 2.62 | 2.72 | 2.38 | 2.79 |
| Cac3 | | 82-96 | ISKISTLGHIKWSSL | 15 | 1669.96 | 2.59 | 2.72 | 2.50 | 2.83 |
| Cac3 | | 82-99 | ISKISTLGHIKWSSLNNF | 18 | 2045.12 | 3.54 | 3.34 | 2.90 | 3.54 |
| Cac3 | | 85-96 | ISTLGHIKWSSL | 12 | 1341.75 | 1.88 | 1.91 | 1.98 | 2.18 |
| Cac3 | | 104-122 | MEFKPENSTRFPSKHLVND | 17 | 2276.11 | 3.02 | 3.09 | 3.11 | 3.33 |
| Cac3 | | 123-146 | ISIFFPNGECNRARYLPQNPDIIA | 21 | 2748.39 | 2.08 | 2.33 | 1.92 | 2.15 |
| Cac3 | | 125-146 | IFFPNGECNRARYLPQNPDIIA | 19 | 2548.28 | 1.25 | 1.48 | 1.24 | 1.52 |
| Cac3 | | 127-146 | FPNGECNRARYLPQNPDIIA | 17 | 2288.12 | 1.48 | 1.68 | 1.18 | 1.47 |
| Cac3 | | 133-153 | NRARYLPQNPDIIAGASSDGA | 19 | 2186.09 | 1.44 | 1.69 | 1.62 | 1.83 |
| Cac3 | | 147-155 | GASSDGAIY | 9 | 840.37 | 0.51 | 0.55 | 0.59 | 0.65 |
| Cac3 | | 181-189 | FGSHGVIQD | 9 | 959.46 | 3.05 | 3.03 | 2.68 | 2.77 |
| Cac3 | | 181-192 | FGSHGVIQDVEA | 12 | 1258.61 | 4.50 | 4.27 | 4.35 | 4.59 |
| Cac3 | | 193-202 | MDTSSADINE | 10 | 1082.43 | 3.63 | 3.49 | 3.58 | 3.79 |
| Cac3 | | 207-212 | AWNLQQ | 6 | 759.38 | 0.81 | 0.83 | 0.75 | 0.82 |
| Cac3 | | 211-216 | QQEALL | 6 | 701.38 | 0.84 | 0.90 | 0.60 | 0.68 |
| Cac3 | | 217-232 | LSSHSNGQVQVWDIKQ | 16 | 1825.92 | 1.06 | 1.23 | 0.98 | 1.19 |
| Cac3 | | 229-242 | DIKQYSHENPIIDL | 13 | 1684.85 | 2.82 | 3.22 | 2.42 | 3.01 |
| Cac3 | | 229-244 | DIKQYSHENPIIDLPL | 14 | 1894.99 | 2.45 | 2.81 | 2.50 | 3.00 |
| Cac3 | | 233-242 | YSHENPIIDL | 9 | 1200.59 | 1.05 | 1.46 | 0.97 | 1.27 |
| Cac3 | | 233-244 | YSHENPIIDLPL | 10 | 1410.73 | 1.07 | 1.36 | 0.93 | 1.23 |
| Cac3 | | 245-256 | VSINSDGTAVND | 12 | 1191.55 | 3.43 | 3.54 | 3.39 | 3.72 |
| Cac3 | | 257-266 | VTWMPTHDSL | 9 | 1186.56 | 1.12 | 1.08 | 1.15 | 1.21 |
| Cac3 | | 279-304 | LDLRTKKEKLQSNREKHDGGVNSCRF | 26 | 3058.60 | 4.24 | 4.06 | 4.58 | 5.04 |
| Cac3 | | 305-312 | NYKNSLIL | 8 | 964.55 | 0.54 | 0.62 | 0.57 | 0.64 |
| Cac3 | | 313-323 | ASADSNGRLNL | 11 | 1117.56 | 0.64 | 0.73 | 0.53 | 0.63 |
| Cac3 | | 324-335 | WDIRNMNKSPIA | 11 | 1444.74 | 2.20 | 2.21 | 2.15 | 2.21 |
| Cac3 | | 324-336 | WDIRNMNKSPIAT | 12 | 1545.78 | 2.84 | 2.85 | 2.79 | 2.96 |
| Cac3 | | 346-352 | LEWSPNF | 6 | 892.42 | 1.53 | 1.51 | 1.49 | 1.74 |
| Cac3 | | 347-352 | EWSPNF | 5 | 779.34 | 1.06 | 1.02 | 0.99 | 1.04 |
| Cac3 | | 357-365 | ATAGQEDGL | 9 | 861.39 | 1.31 | 1.44 | 1.20 | 1.33 |
| Cac3 | | 374-389 | EETIFTHGGHMLGVND | 16 | 1756.80 | 1.08 | 1.26 | 1.18 | 1.29 |
| Cac3 | | 375-389 | ETIFTHGGHMLGVND | 15 | 1627.75 | 0.98 | 1.06 | 1.02 | 1.16 |
| Cac3 | | 376-389 | TIFTHGGHMLGVND | 14 | 1498.71 | 1.01 | 1.09 | 1.05 | 1.19 |
| Cac3 | | 389-399 | DISWDAHDPWL | 10 | 1354.61 | 1.13 | 1.29 | 1.18 | 1.47 |
| Cac3 | | 390-399 | ISWDAHDPWL | 9 | 1239.58 | 0.93 | 1.10 | 0.99 | 1.25 |
| Cac3 | | 390-400 | ISWDAHDPWLM | 10 | 1370.62 | 0.99 | 1.14 | 0.92 | 1.15 |
| Cac3 | | 390-401 | ISWDAHDPWLMC | 11 | 1473.63 | 0.87 | 1.05 | 0.88 | 1.09 |
| Cac3 | | 402-418 | SVANDNSVHIWKPAGNL | 16 | 1821.92 | 1.52 | 1.66 | 1.52 | 1.79 |
| Cac3 | | 404-418 | ANDNSVHIWKPAGNL | 14 | 1635.82 | 1.46 | 1.57 | 1.52 | 1.71 |

**Supplementary file 1B.** Primers used in the studies in yeast

| **Plasmid** | | **Primers** | |
| --- | --- | --- | --- |
| pCac1 | | Forward:  AACCAACCGCGGCCGCGATCTATAGTGTCAGACGGCTTGC  Reverse:  AACCAACTCGAGCGTTTTCATGAATCTACTCG | |
| pCac2 | | Forward:  AACCAATCTAGATAGGGACTGTTATCGAAGG  Reverse:  AACCAACTCGAGCAAAAGGATCCTAAGTTGG | |
| pCac3 | | Forward:  AACCAACGGCCGAATTGGACAACTCCTCCACC  Reverse:  AACCAAGTCGACAGACTGGGAAACCTCGAG | |
| pCac1Δ233-237 | | Forward:  CACAATCCCGTATTGGTAACAGCGATTCTAATACGCCTGTGG  Reverse:  CCACAGGCGTATTAGAATCGCTGTTACCAATACGGGATTGTG | |
| pCac1Δ280-284 | | Forward:  GTGGAGCTGGAAGGTAGCGATGAATTACTGAACAGTAAAG  Reverse:  CTTTACTGTTCAGTAATTCATCGCTACCTTCCAGCTCCAC | |
| pCac1Δ304-322 | | Forward: GTAGTGACGACTTATTGAATTGGTTACAGCAAATGCCTTTGAAGGAG  Reverse: CTCCTTCAAAGGCATTTGCTGTAACCAATTCAATAAGTCGTCACTAC | |
| pCac1Δ340-360 | | Forward:  GATGAGCTCCAATCTTTACTTTATTCTATGGACTTTACTTTGCC  Reverse:  GGCAAAGTAAAGTCCATAGAATAAAGTAAAGATTGGAGCTCATC | |
| pCac1Δ428-432 | | Forward:  GACGGGTTTCTAAGTGATTTGGATGGCCTACCCTGCGCCAAGAG  Reverse:  CCAAATCACTTAGAAACCCGTCAAACTCTCCTTCGCTAGGTACGTC | |
| pCac1K442E/R443E/K444E | | Forward:  GGATGGCCTACCCTGCGCCGAAGAAGAATTTGTAGGCCCATTG  Reverse:  CAATGGGCCTACAAATTCTTCTTCGGCGCAGGGTAGGCCATCC | |
| pCac1Δ463-473 | | Forward: CCTAAAATCCAATTTTGAAAACTTAAAGGCCGAAGTTATCATAGAAACC  Reverse: GGTTTCTATGATAACTTCGGCCTTTAAGTTTTCAAAATTGGATTTTAGG | |
| pCac1Δ497-501 | | Forward:  GAACCCAAAACATCGTCTCTAAATTCCGACTTACAGGCACAGACC  Reverse:  GGTCTGTGCCTGTAAGTCGGAATTTAGAGACGATGTTTTGGGTTC | |
| pCac1S503A | | Forward:  CCAAAAGAAGTAATGCTGACTTACAGGCACAGACCGCTAGCC  Reverse:  GTGCCTGTAAGTCAGCATTACTTCTTTTGGATGGTAGAGACGATG | |
| pCac1S503E | | Forward:  CCAAAAGAAGTAATGAAGACTTACAGGCACAGACCGCTAGCC  Reverse:  GTGCCTGTAAGTCTTCATTACTTCTTTTGGATGGTAGAGACGATG | |
| pCac1S515A | | Forward:  GCCAATCTCAAGCTCCCGAAAAGAAACAAAAAGCAATGATTACAG  Reverse:  CTTTTCGGGAGCTTGAGATTGGCTAGCGGTCTGTGCCTGTAAGTC | |
| pCac1S515E | | Forward:  GCCAATCTCAAGAACCCGAAAAGAAACAAAAAGCAATGATTACAG  Reverse:  CTTTTCGGGTTCTTGAGATTGGCTAGCGGTCTGTGCCTGTAAGTC | |
| pCac1Δ574-584 | | Forward:  GCCATAAGACGCAAATGGGTAATCAAAGACGCACAAAACTGGGAG  Reverse:  CCATTTGCGTCTTATGGCATATTCCTTTATGGTGTTTTTAATTGTTTG | |
| pCac1Δ578-580 | | Forward: GCCATAAGAAGTTCTGGAAAGCCCCGCAAATGGGTAATCAAAGAC  Reverse: GTCTTTGATTACCCATTTGCGGGGCTTTCCAGAACTTCTTATGGC | |
| pCac1Δ575-606 | | Forward:  GAATATGCCATAAGAAGTTAACTTGAACGGATCTTTAGTATATAG  Reverse:  CCGTTCAAGTTAACTTCTTATGGCATATTCCTTTATGGTGTTTTTAATTG | |
| pCac2Δ1-15 | | Forward:  GGCAGGACAATGGTTTACTCGCTCACATTCCAAAAGAACAGTGCG  Reverse:  GAGCGAGTAAACCATTGTCCTGCCCCTTTGCTCTAAGCTTTTCTGC | |
| pCac2E70K | | Forward:  GGCTCGCTGACGCATCACAAACAGGCCATAAATGTAATCCG  Reverse:  CGGATTACATTTATGGCCTGTTTGTGATGCGTCAGCGAGCC | |
| pCac2S206A/A207G | | Forward:  CAGTTTATTCTCTCACAGGCTGGTGACCGGTCTCTGCATGTATATGGAGTC  Reverse:  GTTTATTCTCTCACAGGCTGGTGACCGGTCTCTGCATGTATATG | |
| pCac2V273A/P275A/S276A/G277A | | Forward:  GCTTCACATTTGGCTATCGCTTGGCATGCTGCTGCTCCCGTTGCTTCGCTCACATTCC  Reverse:  GGAATGTGAGCGAAGCAACGGGAGCAGCAGCATGCCAAGCGATAGCCAAATGTGAAGC | |
| pCac2I274A/S276A | | Forward:  GTTTGGTCGTAGCTCCCGCTGGTGTGTATAAGGTGGCTGGTGATG  Reverse:  CTTATACACACCAGCGGGAGCTACGACCAAACCACCACAAGGCG | |
| pCac2D284K/E285K | | Forward:  GTGGCTGGTAAAAAAGTCGCGAACTGCGTATACGTGTATACTAG  Reverse:  CGCAGTTCGCGACTTTTTTACCAGCCACCTTATACACACCACTGG | |
| pCac2R295E | | Forward:  CTGCGTATACGTGTATACTGAATCTGGAATACTGAACAGCG  Reverse:  CGCTGTTCAGTATTCCAGATTCAGTATACACGTATACGCAG | |
| pCac2K306A/N307A/R308A | | Forward:  GCGCTGGTGGCGTTGCTGCTGCTCCTGCGATTAGAATCCC  Reverse:  GGGATTCTAATCGCAGGAGCAGCAGCAACGCCACCAGCGC | |
| pCac2L316A/K318A | | Forward: CCTGCGATTAGAATCCCATCTGCTAAGGCTCCAGCGCTGATGGCG  Reverse: CGCCATCAGCGCTGGAGCCTTAGCAGATGGGATTCTAATCGCAGG | |
| pCac2L316E/K318E | | Forward: CCTGCGATTAGAATCCCATCTGAAAAGGAACCAGCGCTGATGGCG  Reverse: CGCCATCAGCGCTGGTTCCTTTTCAGATGGGATTCTAATCGCAGG | |
| pCac2Δ371-373 | | Forward:  CGTTATGCGTTGTGGGAAATTCACCCATAACTGATTTAG  Reverse:  CTAAATCAGTTATGGGTGAATTTCCCACAACGCATAACG | |
| pCac2M417A/H418A/E420A | | Forward:  GGATAGAGCCGCCAGCGGCTGCTGCAGCTCCACTAGACACTGACG  Reverse:  CGTCAGTGTCTAGTGGAGCTGCAGCAGCCGCTGGCGGCTCTATCC | |
| pCac2Δ425-468 | | Forward:  AGGATAGAGCCGCCAGCGATGCATGCAGAGCCACTAGACACT  Reverse:  AGTGTCTAGTGGCTCTGCATGCATCGCTGGCGGCTCTATCCT | |
| pCac2Δ445-468 | | Forward:  CCAGCGCGAGGCAGGTGGGATCGTGAACATGCTG  Reverse:  CAGCATGTTCACGATCCCACCTGCCTCGCGCTGG | |
| pCac2K447E/K448E | | Forward:  GTGAACATGCTGCCGGTGGAAGAAATCCCCTGCAATAGTAGC  Reverse:  GCTACTATTGCAGGGGATTTCTTCCACCGGCAGCATGTTCAC | |
| pCac2D91KD92K | | Forward:  GACGTCCTGGCGTCTGCGGGCAAGAAGGGCCAAGTGCTGCTATGG  Reverse:  CCATAGCAGCACTTGGCCCTTCTTGCCCGCAGACGCCAGGACGTC | |
| pCac3K284A/K285A/E286E | | Forward:  CTATTAGATCTGAGGACTGCTGCTGCTAAGCTCCAGAGTAACCG  Reverse:  CGGTTACTCTGGAGCTTAGCAGCAGCAGTCCTCAGATCTAATAG | |
| pCac3Δ287-290 | | Forward: CTGGAGGACTAAGCAGAGTAACCGTGAAAAACACGATGGTGGAG  Reverse: GTTACTCTGCTTAGTCCTCAGATCTAATAGGGAGACCGCATTTCC | |
| pCac3Δ306-309 | | Forward: GTAGATTTAACTTAATTCTAGCATCTGCAGATTCAAATGGGAGG  Reverse: GAATTAAGTTAAATCTACAGGAGTTTACTCCACCATCGTGTTTTTC | |
| **Plasmid** | | **Primers** | |
| pCac1 | | Forward:  AACCAACCGCGGCCGCGATCTATAGTGTCAGACGGCTTGC  Reverse:  AACCAACTCGAGCGTTTTCATGAATCTACTCG | |
| pCac2 | | Forward:  AACCAATCTAGATAGGGACTGTTATCGAAGG  Reverse:  AACCAACTCGAGCAAAAGGATCCTAAGTTGG | |
| pCac3 | | Forward:  AACCAACGGCCGAATTGGACAACTCCTCCACC  Reverse:  AACCAAGTCGACAGACTGGGAAACCTCGAG | |
| pCac1Δ233-237 | | Forward:  CACAATCCCGTATTGGTAACAGCGATTCTAATACGCCTGTGG  Reverse:  CCACAGGCGTATTAGAATCGCTGTTACCAATACGGGATTGTG | |
| pCac1Δ280-284 | | Forward:  GTGGAGCTGGAAGGTAGCGATGAATTACTGAACAGTAAAG  Reverse:  CTTTACTGTTCAGTAATTCATCGCTACCTTCCAGCTCCAC | |
| pCac1Δ304-322 | | Forward: GTAGTGACGACTTATTGAATTGGTTACAGCAAATGCCTTTGAAGGAG  Reverse: CTCCTTCAAAGGCATTTGCTGTAACCAATTCAATAAGTCGTCACTAC | |
| pCac1Δ340-360 | | Forward:  GATGAGCTCCAATCTTTACTTTATTCTATGGACTTTACTTTGCC  Reverse:  GGCAAAGTAAAGTCCATAGAATAAAGTAAAGATTGGAGCTCATC | |
| pCac1Δ428-432 | | Forward:  GACGGGTTTCTAAGTGATTTGGATGGCCTACCCTGCGCCAAGAG  Reverse:  CCAAATCACTTAGAAACCCGTCAAACTCTCCTTCGCTAGGTACGTC | |
| pCac1K442E/R443E/K444E | | Forward:  GGATGGCCTACCCTGCGCCGAAGAAGAATTTGTAGGCCCATTG  Reverse:  CAATGGGCCTACAAATTCTTCTTCGGCGCAGGGTAGGCCATCC | |
| pCac1Δ463-473 | | Forward: CCTAAAATCCAATTTTGAAAACTTAAAGGCCGAAGTTATCATAGAAACC  Reverse: GGTTTCTATGATAACTTCGGCCTTTAAGTTTTCAAAATTGGATTTTAGG | |
| pCac1Δ497-501 | | Forward:  GAACCCAAAACATCGTCTCTAAATTCCGACTTACAGGCACAGACC  Reverse:  GGTCTGTGCCTGTAAGTCGGAATTTAGAGACGATGTTTTGGGTTC | |
| pCac1S503A | | Forward:  CCAAAAGAAGTAATGCTGACTTACAGGCACAGACCGCTAGCC  Reverse:  GTGCCTGTAAGTCAGCATTACTTCTTTTGGATGGTAGAGACGATG | |
| pCac1S503E | | Forward:  CCAAAAGAAGTAATGAAGACTTACAGGCACAGACCGCTAGCC  Reverse:  GTGCCTGTAAGTCTTCATTACTTCTTTTGGATGGTAGAGACGATG | |
| pCac1S515A | | Forward:  GCCAATCTCAAGCTCCCGAAAAGAAACAAAAAGCAATGATTACAG  Reverse:  CTTTTCGGGAGCTTGAGATTGGCTAGCGGTCTGTGCCTGTAAGTC | |
| pCac1S515E | | Forward:  GCCAATCTCAAGAACCCGAAAAGAAACAAAAAGCAATGATTACAG  Reverse:  CTTTTCGGGTTCTTGAGATTGGCTAGCGGTCTGTGCCTGTAAGTC | |
| pCac1Δ574-584 | | Forward:  GCCATAAGACGCAAATGGGTAATCAAAGACGCACAAAACTGGGAG  Reverse:  CCATTTGCGTCTTATGGCATATTCCTTTATGGTGTTTTTAATTGTTTG | |
| pCac1Δ578-580 | | Forward: GCCATAAGAAGTTCTGGAAAGCCCCGCAAATGGGTAATCAAAGAC  Reverse: GTCTTTGATTACCCATTTGCGGGGCTTTCCAGAACTTCTTATGGC | |
| pCac1Δ575-606 | | Forward:  GAATATGCCATAAGAAGTTAACTTGAACGGATCTTTAGTATATAG  Reverse:  CCGTTCAAGTTAACTTCTTATGGCATATTCCTTTATGGTGTTTTTAATTG | |
| pCac2Δ1-15 | | Forward:  GGCAGGACAATGGTTTACTCGCTCACATTCCAAAAGAACAGTGCG  Reverse:  GAGCGAGTAAACCATTGTCCTGCCCCTTTGCTCTAAGCTTTTCTGC | |
| pCac2E70K | | Forward:  GGCTCGCTGACGCATCACAAACAGGCCATAAATGTAATCCG  Reverse:  CGGATTACATTTATGGCCTGTTTGTGATGCGTCAGCGAGCC | |
| pCac2S206A/A207G | | Forward:  CAGTTTATTCTCTCACAGGCTGGTGACCGGTCTCTGCATGTATATGGAGTC  Reverse:  GTTTATTCTCTCACAGGCTGGTGACCGGTCTCTGCATGTATATG | |
| pCac2V273A/P275A/S276A/G277A | | Forward:  GCTTCACATTTGGCTATCGCTTGGCATGCTGCTGCTCCCGTTGCTTCGCTCACATTCC  Reverse:  GGAATGTGAGCGAAGCAACGGGAGCAGCAGCATGCCAAGCGATAGCCAAATGTGAAGC | |
| pCac2I274A/S276A | | Forward:  GTTTGGTCGTAGCTCCCGCTGGTGTGTATAAGGTGGCTGGTGATG  Reverse:  CTTATACACACCAGCGGGAGCTACGACCAAACCACCACAAGGCG | |
| pCac2D284K/E285K | | Forward:  GTGGCTGGTAAAAAAGTCGCGAACTGCGTATACGTGTATACTAG  Reverse:  CGCAGTTCGCGACTTTTTTACCAGCCACCTTATACACACCACTGG | |
| pCac2R295E | | Forward:  CTGCGTATACGTGTATACTGAATCTGGAATACTGAACAGCG  Reverse:  CGCTGTTCAGTATTCCAGATTCAGTATACACGTATACGCAG | |
| pCac2K306A/N307A/R308A | | Forward:  GCGCTGGTGGCGTTGCTGCTGCTCCTGCGATTAGAATCCC  Reverse:  GGGATTCTAATCGCAGGAGCAGCAGCAACGCCACCAGCGC | |
| pCac2L316A/K318A | | Forward: CCTGCGATTAGAATCCCATCTGCTAAGGCTCCAGCGCTGATGGCG  Reverse: CGCCATCAGCGCTGGAGCCTTAGCAGATGGGATTCTAATCGCAGG | |
| pCac2L316E/K318E | | Forward: CCTGCGATTAGAATCCCATCTGAAAAGGAACCAGCGCTGATGGCG  Reverse: CGCCATCAGCGCTGGTTCCTTTTCAGATGGGATTCTAATCGCAGG | |
| pCac2Δ371-373 | | Forward:  CGTTATGCGTTGTGGGAAATTCACCCATAACTGATTTAG  Reverse:  CTAAATCAGTTATGGGTGAATTTCCCACAACGCATAACG | |
| pCac2M417A/H418A/E420A | | Forward:  GGATAGAGCCGCCAGCGGCTGCTGCAGCTCCACTAGACACTGACG  Reverse:  CGTCAGTGTCTAGTGGAGCTGCAGCAGCCGCTGGCGGCTCTATCC | |
| pCac2Δ425-468 | | Forward:  AGGATAGAGCCGCCAGCGATGCATGCAGAGCCACTAGACACT  Reverse:  AGTGTCTAGTGGCTCTGCATGCATCGCTGGCGGCTCTATCCT | |
| pCac2Δ445-468 | | Forward:  CCAGCGCGAGGCAGGTGGGATCGTGAACATGCTG  Reverse:  CAGCATGTTCACGATCCCACCTGCCTCGCGCTGG | |
| pCac2K447E/K448E | | Forward:  GTGAACATGCTGCCGGTGGAAGAAATCCCCTGCAATAGTAGC  Reverse:  GCTACTATTGCAGGGGATTTCTTCCACCGGCAGCATGTTCAC | |
| pCac2D91KD92K | | Forward:  GACGTCCTGGCGTCTGCGGGCAAGAAGGGCCAAGTGCTGCTATGG  Reverse:  CCATAGCAGCACTTGGCCCTTCTTGCCCGCAGACGCCAGGACGTC | |
| pCac3K284A/K285A/E286E | | Forward:  CTATTAGATCTGAGGACTGCTGCTGCTAAGCTCCAGAGTAACCG  Reverse:  CGGTTACTCTGGAGCTTAGCAGCAGCAGTCCTCAGATCTAATAG | |
| pCac3Δ287-290 | | Forward: CTGGAGGACTAAGCAGAGTAACCGTGAAAAACACGATGGTGGAG  Reverse: GTTACTCTGCTTAGTCCTCAGATCTAATAGGGAGACCGCATTTCC | |
| pCac3Δ306-309 | | Forward: GTAGATTTAACTTAATTCTAGCATCTGCAGATTCAAATGGGAGG  Reverse: GAATTAAGTTAAATCTACAGGAGTTTACTCCACCATCGTGTTTTTC | |

**Table 1C.** Chemically cross-linked peptides identified by XL-MS.

| **DSS Linkage** | | **CAF-1** | **CAF-1-H3/H4** | **EDC Linkage** | | **CAF-1** | | **CAF-1-H3/H4** |
| --- | --- | --- | --- | --- | --- | --- | --- | --- |
| **Cac1** | **Cac1** |  |  | **Cac1** | **Cac1** | |  |  |
| 100 | 107 |  | ✓ | 1 | 49 | |  | ✓ |
| 136 | 141 | ✓ |  | 2 | 17 | | ✓ | ✓ |
| 144 | 148 | ✓ | ✓ | 2 | 49 | |  | ✓ |
| 144 | 155 |  | ✓ | 6 | 48 | | ✓ |  |
| 148 | 155 | ✓ | ✓ | 12 | 17 | |  | ✓ |
| 148 | 158 | ✓ | ✓ | 13 | 17 | |  | ✓ |
| 155 | 158 |  | ✓ | 17 | 40 | |  | ✓ |
| 158 | 169 | ✓ | ✓ | 17 | 49 | | ✓ |  |
| 187 | 194 | ✓ | ✓ | 33 | 38 | |  | ✓ |
| 194 | 205 |  | ✓ | 33 | 40 | | ✓ | ✓ |
| 202 | 205 | ✓ | ✓ | 33 | 45 | | ✓ | ✓ |
| 205 | 212 | ✓ | ✓ | 33 | 46 | | ✓ | ✓ |
| 212 | 219 | ✓ | ✓ | 33 | 49 | | ✓ | ✓ |
| 235 | 268 |  | ✓ | 37 | 45 | | ✓ | ✓ |
| 236 | 252 | ✓ | ✓ | 37 | 46 | |  | ✓ |
| 268 | 280 | ✓ | ✓ | 37 | 49 | | ✓ |  |
| 270 | 280 | ✓ |  | 45 | 48 | | ✓ |  |
| 280 | 282 | ✓ |  | 62 | 69 | | ✓ |  |
| 313 | 317 |  | ✓ | 73 | 81 | | ✓ | ✓ |
| 489 | 499 |  | ✓ | 86 | 92 | | ✓ | ✓ |
| 499 | 577 | ✓ | ✓ | 118 | 137 | | ✓ | ✓ |
| 521 | 568 | ✓ | ✓ | 118 | 144 | |  | ✓ |
| 521 | 587 | ✓ | ✓ | 118 | 148 | | ✓ | ✓ |
| 553 | 577 | ✓ | ✓ | 118 | 158 | |  | ✓ |
| 553 | 583 | ✓ | ✓ | 131 | 137 | |  | ✓ |
| 568 | 577 | ✓ | ✓ | 136 | 138 | | ✓ | ✓ |
| 568 | 583 | ✓ | ✓ | 153 | 158 | | ✓ |  |
| 577 | 583 | ✓ | ✓ | 153 | 172 | |  | ✓ |
| 577 | 587 | ✓ | ✓ | 154 | 158 | | ✓ | ✓ |
|  |  |  |  | 155 | 159 | | ✓ |  |
|  |  |  |  | 158 | 174 | | ✓ | ✓ |
|  |  |  |  | 158 | 213 | |  | ✓ |
|  |  |  |  | 159 | 166 | | ✓ |  |
|  |  |  |  | 159 | 167 | | ✓ |  |
|  |  |  |  | 165 | 169 | | ✓ |  |
|  |  |  |  | 165 | 172 | | ✓ |  |
|  |  |  |  | 172 | 182 | | ✓ | ✓ |
|  |  |  |  | 174 | 184 | | ✓ | ✓ |
|  |  |  |  | 184 | 188 | |  | ✓ |
|  |  |  |  | 200 | 211 | | ✓ |  |
|  |  |  |  | 205 | 212 | | ✓ |  |
|  |  |  |  | 205 | 213 | | ✓ | ✓ |
|  |  |  |  | 206 | 211 | |  | ✓ |
|  |  |  |  | 208 | 211 | |  | ✓ |
|  |  |  |  | 211 | 213 | |  | ✓ |
|  |  |  |  | 211 | 220 | | ✓ | ✓ |
|  |  |  |  | 211 | 221 | | ✓ | ✓ |
|  |  |  |  | 211 | 261 | | ✓ | ✓ |
|  |  |  |  | 211 | 275 | | ✓ | ✓ |
|  |  |  |  | 212 | 221 | | ✓ | ✓ |
|  |  |  |  | 213 | 219 | | ✓ |  |
|  |  |  |  | 213 | 223 | | ✓ | ✓ |
|  |  |  |  | 213 | 235 | | ✓ | ✓ |
|  |  |  |  | 213 | 236 | | ✓ |  |
|  |  |  |  | 213 | 252 | | ✓ |  |
|  |  |  |  | 213 | 273 | |  | ✓ |
|  |  |  |  | 214 | 223 | | ✓ |  |
|  |  |  |  | 214 | 235 | | ✓ |  |
|  |  |  |  | 215 | 219 | |  | ✓ |
|  |  |  |  | 215 | 223 | | ✓ | ✓ |
|  |  |  |  | 219 | 261 | | ✓ | ✓ |
|  |  |  |  | 219 | 275 | | ✓ | ✓ |
|  |  |  |  | 220 | 235 | | ✓ |  |
|  |  |  |  | 220 | 236 | | ✓ | ✓ |
|  |  |  |  | 221 | 235 | |  | ✓ |
|  |  |  |  | 223 | 246 | |  | ✓ |
|  |  |  |  | 223 | 275 | | ✓ | ✓ |
|  |  |  |  | 224 | 235 | | ✓ |  |
|  |  |  |  | 235 | 239 | | ✓ | ✓ |
|  |  |  |  | 235 | 246 | | ✓ | ✓ |
|  |  |  |  | 235 | 249 | | ✓ |  |
|  |  |  |  | 235 | 251 | | ✓ |  |
|  |  |  |  | 235 | 261 | | ✓ |  |
|  |  |  |  | 235 | 275 | | ✓ |  |
|  |  |  |  | 235 | 277 | | ✓ |  |
|  |  |  |  | 239 | 252 | | ✓ |  |
|  |  |  |  | 239 | 270 | | ✓ |  |
|  |  |  |  | 239 | 273 | | ✓ |  |
|  |  |  |  | 246 | 252 | | ✓ | ✓ |
|  |  |  |  | 246 | 273 | | ✓ |  |
|  |  |  |  | 249 | 260 | | ✓ | ✓ |
|  |  |  |  | 249 | 268 | | ✓ |  |
|  |  |  |  | 252 | 261 | | ✓ |  |
|  |  |  |  | 252 | 334 | | ✓ | ✓ |
|  |  |  |  | 260 | 275 | | ✓ | ✓ |
|  |  |  |  | 261 | 268 | | ✓ | ✓ |
|  |  |  |  | 261 | 270 | | ✓ | ✓ |
|  |  |  |  | 261 | 273 | | ✓ |  |
|  |  |  |  | 268 | 277 | | ✓ |  |
|  |  |  |  | 268 | 285 | |  | ✓ |
|  |  |  |  | 268 | 286 | | ✓ |  |
|  |  |  |  | 270 | 275 | | ✓ | ✓ |
|  |  |  |  | 270 | 284 | | ✓ |  |
|  |  |  |  | 270 | 285 | | ✓ | ✓ |
|  |  |  |  | 273 | 284 | | ✓ | ✓ |
|  |  |  |  | 273 | 285 | | ✓ |  |
|  |  |  |  | 273 | 286 | | ✓ | ✓ |
|  |  |  |  | 275 | 282 | | ✓ | ✓ |
|  |  |  |  | 275 | 293 | | ✓ |  |
|  |  |  |  | 275 | 317 | |  | ✓ |
|  |  |  |  | 277 | 282 | | ✓ | ✓ |
|  |  |  |  | 280 | 284 | | ✓ |  |
|  |  |  |  | 280 | 297 | |  | ✓ |
|  |  |  |  | 282 | 286 | | ✓ |  |
|  |  |  |  | 282 | 292 | | ✓ | ✓ |
|  |  |  |  | 282 | 297 | | ✓ | ✓ |
|  |  |  |  | 282 | 298 | | ✓ | ✓ |
|  |  |  |  | 284 | 293 | |  | ✓ |
|  |  |  |  | 285 | 313 | |  | ✓ |
|  |  |  |  | 291 | 297 | | ✓ | ✓ |
|  |  |  |  | 297 | 313 | |  | ✓ |
|  |  |  |  | 317 | 332 | | ✓ | ✓ |
|  |  |  |  | 320 | 328 | | ✓ | ✓ |
|  |  |  |  | 328 | 332 | | ✓ | ✓ |
|  |  |  |  | 328 | 334 | | ✓ | ✓ |
|  |  |  |  | 460 | 583 | | ✓ |  |
|  |  |  |  | 480 | 492 | | ✓ |  |
|  |  |  |  | 504 | 564 | |  | ✓ |
|  |  |  |  | 504 | 568 | |  | ✓ |
|  |  |  |  | 504 | 577 | | ✓ |  |
|  |  |  |  | 519 | 569 | | ✓ |  |
|  |  |  |  | 521 | 539 | | ✓ |  |
|  |  |  |  | 521 | 569 | | ✓ | ✓ |
|  |  |  |  | 521 | 579 | | ✓ |  |
|  |  |  |  | 529 | 553 | | ✓ |  |
|  |  |  |  | 529 | 564 | |  | ✓ |
|  |  |  |  | 535 | 553 | | ✓ | ✓ |
|  |  |  |  | 535 | 560 | |  | ✓ |
|  |  |  |  | 535 | 564 | | ✓ |  |
|  |  |  |  | 539 | 560 | | ✓ |  |
|  |  |  |  | 539 | 583 | | ✓ | ✓ |
|  |  |  |  | 560 | 569 | | ✓ | ✓ |
|  |  |  |  | 564 | 569 | | ✓ | ✓ |
|  |  |  |  | 564 | 588 | |  | ✓ |
|  |  |  |  | 564 | 593 | | ✓ |  |
|  |  |  |  | 568 | 579 | | ✓ | ✓ |
|  |  |  |  | 568 | 588 | | ✓ | ✓ |
|  |  |  |  | 568 | 593 | | ✓ | ✓ |
|  |  |  |  | 569 | 577 | | ✓ | ✓ |
|  |  |  |  | 569 | 583 | | ✓ | ✓ |
|  |  |  |  | 569 | 587 | | ✓ | ✓ |
|  |  |  |  | 577 | 588 | | ✓ | ✓ |
|  |  |  |  | 577 | 593 | | ✓ | ✓ |
|  |  |  |  | 579 | 583 | | ✓ | ✓ |
|  |  |  |  | 583 | 588 | | ✓ | ✓ |
|  |  |  |  | 583 | 593 | | ✓ | ✓ |
|  |  |  |  |  |  | |  |  |
| **Cac2** | **Cac2** |  |  | **Cac2** | **Cac2** | |  |  |
| 29 | 43 | ✓ | ✓ | 28 | 38 | | ✓ |  |
|  |  |  |  | 36 | 43 | |  | ✓ |
|  |  |  |  | 47 | 57 | | ✓ |  |
|  |  |  |  | 232 | 237 | | ✓ | ✓ |
|  |  |  |  | 232 | 243 | |  |  |
|  |  |  |  | 447 | 455 | | ✓ |  |
|  |  |  |  |  |  | |  |  |
|  |  |  |  | **Cac3** | **Cac3** | |  |  |
|  |  |  |  | 21 | 28 | | ✓ |  |
|  |  |  |  | 285 | 296 | | ✓ |  |
|  |  |  |  | 286 | 293 | | ✓ |  |
|  |  |  |  | 287 | 296 | | ✓ |  |
|  |  |  |  |  |  | |  |  |
| **Cac1** | **Cac2** |  |  | **Cac1** | **Cac2** | |  |  |
| 499 | 447 | ✓ | ✓ | 330 | 59 | |  | ✓ |
|  |  |  |  | 92 | 91 | | ✓ |  |
|  |  |  |  | 158 | 91 | | ✓ |  |
|  |  |  |  | 172 | 91 | | ✓ |  |
|  |  |  |  | 148 | 285 | | ✓ |  |
|  |  |  |  | 158 | 285 | | ✓ |  |
|  |  |  |  | 568 | 284 | | ✓ | ✓ |
|  |  |  |  | 521 | 436 | |  | ✓ |
|  |  |  |  |  |  | |  |  |
| **Cac1** | **Cac3** |  |  | **Cac1** | **Cac3** | |  |  |
| 282 | 307 | ✓ | ✓ | 593 | 28 | | ✓ |  |
| 235 | 287 |  | ✓ |  |  | |  |  |
|  |  |  |  |  |  | |  |  |
| **H3** | **H3** |  |  | **H3** | **H3** | |  |  |
| 4 | 9 |  | ✓ | 4 | 77 | |  | ✓ |
| 4 | 14 |  | ✓ | 23 | 59 | |  | ✓ |
| 4 | 18 |  | ✓ | 27 | 73 | |  | ✓ |
| 4 | 56 |  | ✓ | 36 | 59 | |  | ✓ |
| 4 | 64 |  | ✓ | 37 | 73 | |  | ✓ |
| 4 | 115 |  | ✓ | 56 | 73 | |  | ✓ |
| 4 | 122 |  | ✓ | 56 | 123 | |  | ✓ |
| 9 | 18 |  | ✓ | 59 | 64 | |  | ✓ |
| 9 | 64 |  | ✓ | 59 | 122 | |  | ✓ |
| 9 | 79 |  | ✓ | 64 | 73 | |  | ✓ |
| 9 | 122 |  | ✓ | 64 | 77 | |  | ✓ |
| 14 | 18 |  | ✓ | 64 | 123 | |  | ✓ |
| 14 | 23 |  | ✓ |  |  | |  |  |
| 14 | 56 |  | ✓ |  |  | |  |  |
| 14 | 122 |  | ✓ |  |  | |  |  |
| 18 | 27 |  | ✓ |  |  | |  |  |
| 18 | 56 |  | ✓ |  |  | |  |  |
| 18 | 64 |  | ✓ |  |  | |  |  |
| 18 | 79 |  | ✓ |  |  | |  |  |
| 18 | 122 |  | ✓ |  |  | |  |  |
| 23 | 27 |  | ✓ |  |  | |  |  |
| 23 | 56 |  | ✓ |  |  | |  |  |
| 23 | 64 |  | ✓ |  |  | |  |  |
| 27 | 37 |  | ✓ |  |  | |  |  |
| 27 | 56 |  | ✓ |  |  | |  |  |
| 27 | 122 |  | ✓ |  |  | |  |  |
| 37 | 56 |  | ✓ |  |  | |  |  |
| 56 | 64 |  | ✓ |  |  | |  |  |
| 56 | 79 |  | ✓ |  |  | |  |  |
| 56 | 115 |  | ✓ |  |  | |  |  |
| 56 | 122 |  | ✓ |  |  | |  |  |
| 64 | 79 |  | ✓ |  |  | |  |  |
| 64 | 122 |  | ✓ |  |  | |  |  |
| 79 | 122 |  | ✓ |  |  | |  |  |
| 115 | 122 |  | ✓ |  |  | |  |  |
|  |  |  |  |  |  | |  |  |
| **H4** | **H4** |  |  | **H4** | **H4** | |  |  |
| 5 | 12 |  | ✓ | 8 | 52 | |  | ✓ |
| 5 | 20 |  | ✓ | 8 | 53 | |  | ✓ |
| 12 | 20 |  | ✓ | 12 | 53 | |  | ✓ |
| 16 | 20 |  | ✓ | 20 | 24 | |  | ✓ |
| 16 | 31 |  | ✓ | 20 | 52 | |  | ✓ |
| 20 | 31 |  | ✓ | 20 | 53 | |  | ✓ |
| 31 | 59 |  | ✓ | 52 | 59 | |  | ✓ |
| 31 | 91 |  | ✓ | 53 | 59 | |  | ✓ |
|  |  |  |  | 79 | 85 | |  | ✓ |
|  |  |  |  |  |  | |  |  |
| **H3** | **H4** |  |  | **H3** | **H4** | |  |  |
| 3 | 20 |  | ✓ | 18 | 102 | |  | ✓ |
| 4 | 20 |  | ✓ | 37 | 52 | |  | ✓ |
| 4 | 31 |  | ✓ | 37 | 53 | |  | ✓ |
| 9 | 20 |  | ✓ | 56 | 102 | |  | ✓ |
| 56 | 20 |  | ✓ | 59 | 31 | |  | ✓ |
| 56 | 31 |  | ✓ | 59 | 79 | |  | ✓ |
| 64 | 20 |  | ✓ | 64 | 24 | |  | ✓ |
| 64 | 31 |  | ✓ | 64 | 52 | |  | ✓ |
| 64 | 44 |  | ✓ | 64 | 53 | |  | ✓ |
| 79 | 20 |  | ✓ | 64 | 102 | |  | ✓ |
| 79 | 31 |  | ✓ | 73 | 8 | |  | ✓ |
| 79 | 79 |  | ✓ | 73 | 59 | |  | ✓ |
| 122 | 31 |  | ✓ | 79 | 52 | |  | ✓ |
| 122 | 59 |  | ✓ | 79 | 74 | |  | ✓ |
|  |  |  |  | 81 | 12 | |  | ✓ |
|  |  |  |  | 81 | 79 | |  | ✓ |
|  |  |  |  | 122 | 53 | |  | ✓ |
|  |  |  |  | 123 | 59 | |  | ✓ |
|  |  |  |  |  |  | |  |  |
| **Cac1** | **H3** |  |  | **Cac1** | **H3** | |  |  |
| 577 | 4 |  | ✓ | 579 | 18 | |  | ✓ |
| 583 | 4 |  | ✓ | 593 | 18 | |  | ✓ |
| 444 | 64 |  | ✓ | 444 | 59 | |  | ✓ |
| 577 | 64 |  | ✓ |  |  | |  |  |
| 577 | 122 |  | ✓ |  |  | |  |  |
|  |  |  |  |  |  | |  |  |
|  |  |  |  | **Cac3** | **H3** | |  |  |
|  |  |  |  | 7 | 9 | |  | ✓ |
|  |  |  |  | 21 | 18 | |  | ✓ |
|  |  |  |  |  |  | |  |  |
| **Cac1** | **H4** |  |  | **Cac1** | **H4** | |  |  |
| 194 | 20 |  | ✓ | 588 | 8 | |  | ✓ |
| 577 | 20 |  | ✓ | 579 | 20 | |  | ✓ |
| 577 | 31 |  | ✓ | 317 | 53 | |  | ✓ |
| 577 | 79 |  | ✓ | 464 | 59 | |  | ✓ |
|  |  |  |  | 569 | 79 | |  | ✓ |

**Table 1D**. Cac1C putative dimer contacts

| Source atom | |  | Target atom  (*symmetry mate) | | Distance  (Å) | Potential H-bonds |
| --- | --- | --- | --- | --- | --- | --- |
| I572 | CG1 | – | *Q590 | OE1 | 3.98 |  |
| I572 | CG1 | – | *Q590 | NE2 | 3.76 |  |
| I572 | CD1 | – | *Q590 | NE2 | 3.74 |  |
| K577 | CG | – | *A597 | O | 3.86 |  |
| K577 | CG | – | *A597 | CB | 3.91 |  |
| K577 | NZ | – | *A597 | CB | 3.63 |  |
| D579 | OD2 | – | *N600 | C | 3.35 |  |
| D579 | OD2 | – | *N600 | O | 3.42 | + |
| L580 | CD2 | – | *E593 | OE2 | 3.36 |  |
| K583 | CE | – | *Q593 | OE2 | 3.73 |  |
| K583 | CE | – | *Q593 | CD | 3.92 |  |
| K583 | CE | – | *Q593 | OE1 | 3.59 |  |
| K583 | NZ | – | *R596 | NH1 | 3.80 | + |
| I586 | O | – | *A589 | CB | 3.06 |  |
| K587 | C | – | *A589 | N | 3.92 |  |
| K587 | O | – | *K587 | O | 3.42 | + |
| K587 | O | – | *A589 | CB | 3.99 |  |
| K587 | O | – | *A589 | N | 2.90 | +++ |
| K587 | O | – | *A589 | CA | 3.88 |  |
| K587 | O | – | *Q590 | N | 3.77 | + |
| K587 | O | – | *D588 | CA | 3.36 |  |
| K587 | O | – | *D588 | C | 3.50 |  |
| K587 | O | – | *D588 | OD1 | 3.42 | + |
| K587 | CD | – | *Q590 | OE1 | 3.87 |  |
| Q552 | NE2 | – | WAT | O | 3.27 | +++ |
| N559 | CA | – | WAT | O | 3.77 |  |
| N559 | CB | – | WAT | O | 3.89 |  |
| K560 | N | – | WAT | O | 3.48 | + |

*The reciprocal symmetry related contacts are not shown
